# Editorial: Alloplastic materials and emerging technologies in reconstructive and plastic surgery: an evidence based state-of-the-art

**DOI:** 10.3389/fsurg.2026.2007970

**Published:** 2026-10-22

**Authors:** Giuseppe Cottone

**Affiliations:** 1 IRCCS Ospedale Galeazzi-Sant'Ambrogio, Milano, Italy; 2 Policlinico San Pietro, Ponte San Pietro, Italy; 3 Casa di Cura Privata Villa Sant'Apollonia, Bergamo, Italy

**Keywords:** alloplastic biomaterials, emerging technologies, evidence-based, minimally invasive, plastic surgery, reconstructive surgery, tissue engineering

## Introduction

Plastic and reconstructive surgery has always evolved at the interface between surgical ingenuity and technological innovation. Few other surgical disciplines have adopted new materials, devices, alloplastic substitutes, injectable biomaterials, and minimally invasive technologies at such a rapid pace. From the reconstruction of complex tissue defects to the modulation of tissue quality and contour, emerging technologies are progressively expanding the boundaries of what can be achieved for the benefit of our patients.

Yet, as innovation accelerates, a fundamental question becomes increasingly difficult to ignore: how quickly should a promising technology move from the operating room to established clinical practice?

The Research Topic “*Alloplastic Materials and Emerging Technologies in Reconstructive and Plastic Surgery: An Evidence-Based State of the Art”* stemmed from and revolves around this question. The published contributions provide a heterogeneous but remarkably coherent cross-section of the current landscape, encompassing tissue substitutes, bioresorbable meshes, biostimulatory materials, and novel minimally invasive technologies. More importantly, they illustrate different stages of the journey from innovation to evidence: from the initial demonstration of technical feasibility to prospective clinical investigation and validation.

Rather than defining a definitive state-of-the-art, these studies reveal both the promise of ongoing biomaterial research and the clear gaps that still separate technological novelty from established clinical value.

### Ideas generate hypotheses: the importance of biological integration

The history of biomaterials in reconstructive surgery has progressively moved beyond the concept of mechanical replacement. Biomaterials are now intended to act as active participants in the tissue regenerative process, interacting with the host environment and ultimately becoming part of a dynamic yet stable biological reconstruction.

The report by Gabar et al. is examplary. Although limited by its case-report nature, this work highlights the potential of skin substitutes to expand the reconstructive arsenal, particularly in challenging wound environments.

Its deeper significance lies not in defining a new standard of care, but in demonstrating how a biomaterial can be integrated into established reconstructive principles to address specific clinical problems. Such reports are often where innovation begins. They generate hypotheses, identify potential applications, and provide the rationale for more systematic investigation.

A similar concept emerges from the report by Putko et al. This case raises a particularly intriguing question: does a durable reconstruction necessarily require a durable implant?

The answer, again, is based on our understanding of tissue-material interactions rather than on the persistence of the implant itself. A bioresorbable scaffold may provide temporary mechanical support while allowing progressive biological integration and replacement by functional host tissue. The clinical appeal of this concept is considerable, particularly in situations where a permanent foreign body may be undesirable. Nevertheless, a single successful case cannot establish comparative superiority; rather, it generates a hypohesis that warrants further investigation.

Together, these reports illustrate a fundamental principle: the most interesting biomaterials are not necessarily those that remain in the body, but those that can significantly influence what the body does after implantation.

### Increasing methodological sophistication

The transition from proof of concept to evidence generation is exemplified by the prospective study of Bravo et al. The value of this study lies not simply in the evaluation of a class of injectable biomaterials, but also in its attempt to assess their effects across multiple dimensions. Clinical assessment was complemented by ultrasonography, biophysical measurements, and histological analysis, providing a more comprehensive picture of tissue response than subjective aesthetic evaluation alone.

Both formulations were associated with quantitative and qualitative improvements. These findings support the biological activity of PLLA-based treatments while simultaneously illustrating the limitations of early-stage clinical research: the relatively small cohort and short follow-up preclude conclusions regarding long-term durability and sustained clinical benefit. This distinction is crucial. In aesthetic and reconstructive surgery, an early measurable change is not necessarily associated with a clinically significant long-term outcome. This field will require an increasing number of studies capable of linking biological effects, objective measurement, patient perception, and durability within the same investigative framework.

The aforementioned study represents an important step in that direction. It also suggests a broader methodological evolution: as biomaterials become increasingly sophisticated, the methods used to evaluate them must become equally sophisticated.

### The paradox of evidence accumulation

If the prospective study illustrates the progressive refinement of evidence, the meta-analysis by Zhou and Zhuang highlights a different and equally important issue: more evidence does not necessarily translate into greater scientific certainty.

This analysis provides a quantitative perspective on the safety profile of an increasingly widespread minimally invasive aesthetic procedure. At the same time, substantial heterogeneity across outcomes underscores the difficulty of interpretation when the underlying literature is methodologically and clinically diverse.

A meta-analysis can synthesize available evidence, but it cannot compensate for fundamental inconsistencies in how that evidence was generated.

The lesson extends well beyond the topic of thread lifting: as new technologies proliferate, the field must resist the temptation to equate the volume of published literature with the strenght of evidence. Evidence becomes clinically meaningful when studies are sufficiently comparable, transparent, reproducible, and methodologically rigorous to allow reliable interpretation.

### The translational gap

These four contributions reveal an evidence continuum rather than a hierarchy of studies.

A case report can establish feasibility and generate a hypothesis. A long-term observational study can provide insights into durability and tissue-material interactions. A prospective controlled investigation can introduce predefined aims and comparative outcome assessment. A systematic review and meta-analysis can integrate findings across multiple studies and identify patterns that may not be apparent at the lower levels of the evidence hierarchy. Each stage also exposes the limitations of the preceding one.

This continuum is particularly relevant to plastic surgery, where technological adoption can sometimes outpace formal clinical validation. A fascinating new tool, an attractive early result, or a technically elegant procedure may rapidly generate enthusiasm. However, clinical enthusiasm and scientific certainty are not interchangeable.

The challenge, therefore, is not to slow innovation, but to build a more efficient bridge between innovation and evidence.

Such a bridge requires prospective investigation, standardized outcomes, adequate follow-up, transparent reporting of complications, and, whenever possible, multicentre collaboration. It also requires attention to outcomes that extend beyond conventional surgical endpoints. Patient-reported outcomes, quality of life, functional recovery, aesthetic satisfaction, and long-term tissue behaviour should increasingly be considered alongside traditional measures of morbidity and technical success.

In this context, evidence-based medicine should not be regarded as an obstacle to innovation, but rather as a mechanism through which promising innovations acquire strong clinical value.

### From material-centered to patient-centered innovation

A common theme across these contributions is the need to reconsider what we mean when we talk about “technological success”.

A new material should not be applauded simply because it is novel, bioresorbable, minimally invasive, or technologically sophisticated. Rather, it should be welcomed because it addresses a significant clinical problem better—or differently enough—to justify its use.

This shifts the focus from material-centered to patient-centered innovation.

For biomaterials and emerging technologies, the relevant questions extend beyond their intriguing properties or biological activity. Do they improve outcomes that matter to patients? Do they reduce morbidity? Do they provide long-term tissue integration? Are they versatile across different clinical settings? Is their risk-benefit profile acceptable? And, perhaps more importantly, can their advantages be demonstrated against existing alternatives?

### What comes next?

Future surgical innovation will likely emerge from the convergence of biomaterials research, tissue engineering, digital technologies, personalized medicine, and advanced manufacturing. Patient-specific implants, bioactive and bioresorbable scaffolds, 3D printing and bioprinting, and novel minimally invasive technologies are already reshaping the field of tissue reconstruction and modulation.

But technological sophistication alone will not determine which of these approaches will become clinically significant.

The future will depend on our ability to link innovation with rigorous evaluation. High-quality scientific literature will be essential. Dedicated registries may also become particularly important for emerging technologies that are rapidly adopted but difficult to investigate systematically. Equally important will be collaboration: the most significant advances are unlikely to emerge from surgery, engineering, or industry in isolation, but rather from their intersection.

## Conclusions

The works included in this Research Topic provide neither a final answer nor a definitive endpoint. Instead, they offer a snapshot of a field in transition.

They demonstrate how contemporary plastic and reconstructive surgery is moving from simple tissue replacement toward a more sophisticated understanding of material–tissue interactions, biological integration, personalized reconstruction, and minimally invasive modulation. At the same time, they remind us that the path from a novel idea to established clinical practice is necessarily incremental and must rely on robust scientific validation.

Perhaps this is the most important message emerging from this Editorial. The future of alloplastic materials and emerging technologies will not be defined by how rapidly new solutions can be introduced, but by how rigorously we can demonstrate which solutions provide significant, reproducible, and long-term benefits to our patients.

Innovation should therefore not be viewed as being in opposition to evidence: the two are complementary stages of the same translational process.

The surgeon's task is to remain sufficiently curious to embrace new possibilities, yet sufficiently critical to demand evidence before declaring them established.

Innovation opens the door; evidence determines whether we should walk through it.

